# Walking or breathing: comparing the 6-minute walking distance test to the pulmonary function test for lung resection candidates

**DOI:** 10.34172/jcvtr.31816

**Published:** 2024-06-25

**Authors:** Ali Mehri, Fariba Zabihi, Taha Sharafian, Mona Kabiri, Reza Rezaei

**Affiliations:** ^1^Endoscopic and Minimally Invasive Surgery Research Center, Mashhad University of Medical Sciences, Mashhad, Iran; ^2^Department of General Surgery, Mashhad University of Medical Sciences, Mashhad, Iran; ^3^Faculty of Medicine, Mashhad University of Medical Sciences, Mashhad, Iran; ^4^Department of Pharmaceutical Nanotechnology, School of Pharmacy, Mashhad University of Medical Sciences, Mashhad, Iran; ^5^Clinical Research Development Unit, Ghaem Hospital, Mashhad University of Medical Sciences, Mashhad, Iran; ^6^Department of Thoracic Surgery, Endoscopic and Minimally Invasive Surgery Research Center, Mashhad University of Medical Sciences, Mashhad, Iran

**Keywords:** Thoracic surgery, Spirometry, Lung resection, 6MWD, PFT

## Abstract

**Introduction:**

Given the limited use of the 6-minute walking distance (6MWD) test as a replacement for standard tests in thoracic surgery, insufficient research exists on the prognostic value of this test, and further studies are necessary. This study aimed to investigate the correlation between pulmonary function tests (PFT) and the 6MWD test in lung resection patients.

**Methods:**

This cross-sectional study, conducted in 2021-2022, involved lung resection candidates referred to the thoracic surgery clinic. Demographic data, including age, sex, and body mass index (BMI), were collected, and pulmonary function tests and 6MWD tests were conducted for all patients. The sample size of the study was 31, and all patients received routine treatment during hospitalization.

**Results:**

Of the 31 subjects included in the study, 16 were male (51.6%) and 15 (48.4%) were female. The mean age of the patients was 33.45±13.78 years. The median forced expiratory volume in one second (FEV1) and the mean ratio of FEV1/forced vital capacity (FVC) were 2.16 (1.49–2.85) liters and 81.80±7.34%, respectively. No significant correlation was found between the results of 6MWD and PFT, including FVC, FEV1, and FEV1/FVC ratio (*P*>0.05).

**Conclusion:**

The 6MWD test is a more economical and easily accessible test than PFT. However, this study found no correlation between the 6MWD test and spirometry parameters. Therefore, we suggest that surgeons should not rely on the 6MWD test as a predictive value for assessing respiratory function in lung resection candidates. The study’s findings have important implications for clinical practice.

## Introduction

 A patient who undergoes a thoracotomy and lung resection experiences significant physiological changes. Resection of the lung in patients with reduced capacities, such as those who suffer from chronic obstructive pulmonary disease (COPD), may cause respiratory failure.^1,2^ The risks associated with critical surgical procedures, such as thoracic surgery, increase the likelihood of patient mortality when underlying conditions exist. On the other hand, the prevalence of pulmonary complications following surgery is greater than that of cardiac problems; they extend hospital stays by an average of one to two weeks, and they undoubtedly contribute to the mortality rate of surgery.^3^ Therefore, an assessment of the patient’s condition is necessary before lung resection and thoracotomy are performed.

 A pulmonary function test (PFT) is used in a non-clinical setting to evaluate the respiratory system. For several decades, the PFT has been essential in the assessment of lung resection candidates. The forced vital capacity (FVC) and the forced expiratory volume in 1 second (FEV1) are relevant markers. It has been demonstrated in several studies that these measures help identify complications and patient mortality^4^ and are effective predictors of the patient’s status following surgery.^5^ However, this test is notable for the possibility of contaminating the spirometry device with respiratory pathogens such as tuberculosis and COVID-19.

 Additionally, despite observing standards, some patients refuse to take the test for fear of contracting COVID-19. Furthermore, this test is not feasible in conditions such as Bell’s palsy, where the patient cannot blow into the tube.^6^ Several requirements and costs are associated with performing spirometry, including the availability of the necessary equipment and facilities and additional fees, which can be problematic if the equipment malfunctions or the facilities are inaccessible. Due to these circumstances, the 6-minute walking distance (6MWD) test has been recommended as an alternative method of assessing respiratory function.^7,8^ The 6MWD test is a non-invasive test to evaluate an individual’s cardiopulmonary function by measuring the distance walked over a given time.^9^ The test has also demonstrated efficacy in estimating mortality and morbidity in individuals with COPD before surgery.^10,11^ This study aims to investigate the correlation between the spirometric respiratory function test and the six-minute walking distance test in lung resection candidates.

## Materials and Methods

###  Study design

 This cross-sectional study was conducted in 2021 on lung resection candidates referred to the thoracic surgery department of Ghaem Hospital, Mashhad University of Medical Sciences, Mashhad, Iran. Candidate patients for lung resection who consented to participate in the study were included. Exclusion criteria were: (i) unwillingness to continue the study; (ii) unstable angina during the previous month; (iii) heart attack during the previous month; (iv) resting heart rate over 120; (v) systolic blood pressure over 180 mmHg; and (vi) diastolic blood pressure over 100 mmHg. As soon as the patients were included, demographic information, including age, gender, and body mass index (BMI), was collected. PFT and 6MWD tests were performed before surgery, and necessary treatment procedures were performed for all patients during hospitalization.The 6MWD test measures the distance a patient can walk within a span of 6 minutes, using meters as the unit of measurement. It is hypothesized that this test can provide an estimation of pulmonary function.

###  Sample size

 The sample size (n = 31) was determined based on a previous study by Agrawal et al,^9^ which reported a correlation coefficient of 6MWD and FEV1 (r = 0.57). A 0.05 alpha error and 90% power (beta = 0.1) were considered.

###  Statistical analysis

 The data were analyzed using SPSS version 22 and involved descriptive and analytical statistics. Quantitative data normality was checked using the Kolmogorov-Smirnov test. Mean ± SD or median (percentile 25-75) was used to describe normally or non-normally distributed variables, respectively. Independent sample t-test/Mann-Whitney test compared quantitative variables by gender. Qualitative variables were assessed using Chi-square/Fisher’s exact test. Pearson’s test/Spearman’s non-parametric test determined correlations between variables. Different tests were used for quantitative and qualitative variables, and *P*< 0.05 was considered significant.

## Results

 A total of 31 patients were enrolled in the study, with 16 (51.6%) being male and 15 (48.4%) female. The mean age of the patients was 33.45 ± 13.78 years, ranging from 17 to 66 years. The mean BMI was 23.61 ± 4.29 kg/m^2^, with a range of 15.88 to 36.32 kg/m^2^. The most common reason for surgery was a hydatid cyst (64.51%), followed by local bronchiectasis and empyema (9.6% each). There were two cases of pneumothorax (6.45%) and one case of lung abscess (3.22%).

Table 1 displays the results of PFT and the 6MWD test. The mean FEV1 and FEV1/FVC ratio were 2.24 ± 0.92 liters and 81.80 ± 7.34%, respectively.

**Table 1:** The statistical description of the 6MWD test and pulmonary function test indices. Mean ± SD was used to describe continuous data with normal distribution, and median (percentile 25-75) was used for non-normal distribution. Frequency (%) was described for qualitative variables

**Characteristic**	**Total ** **(n=31)**	**Groups**		***P*** ** Value**
		**Female(n=15) **	**Male(n=16)**	
Age(year)	33.45 ± 13.78	35.80 ± 16.38	31.25 ± 10.91	0.375^†^
BMI (kg/m2)	23.56 ± 4.25	24.30 ± 5.09	22.87 ± 3.3	0.364^†^
Low weight (BMI < 18.5)	4 (12.9)	2 (13.3)	2 (12.5)	0.576^†^
Normal (BMI 18.5-25)	15 (48.4)	6 (40.0)	9 (56.3)	
High weight (BMI 25-30)	10 (32.3)	5 (33.3)	5 (31.3)	
Obesity (BMI > 30)	2 (6.5)	2 (13.3)	0 (0.0)	
6MWD (m)	363.00 (318.00 – 442.00)	358.50 (325.00 – 406.00)	416.00 (313.50 – 448.50)	0.495^¶^
FEV1 (L)	2.16 (1.49 – 2.85 )	1.66 (1.40 – 2.56)	2.17 (1.80 – 3.15)	0.140^¶^
FVC (L)	2.68 (1.90 – 3.41)	1.93 (1.79 – 3.04)	2.78 (2.03 – 3.54)	0.129^¶^
FEV1/FVC (%)	81.80 ± 7.34	80.96 ± 7.42	82.60 ± 7.41	0.542^†^

SD: standard deviation; 6MWD: 6-minute walking distance; FEV1: forced expiratory volume in the first second; FVC: forced vital capacity; ¶: Mann-Whitney test; †: Independent sample t-test.

 In Table 2 the correlation between age, body mass index, FEV1 and FEV1/FVC is investigated. As it is known, there is no relationship between spirometric indices and 6MWD.

**Table 2:** The correlation of age, body mass index, FEV1 and FEV1/FVC

**Characteristic **	**Age (Year)**		**BMI (kg/m2)**		**FEV1(L)**		**FVC (L)**		**FEV1/FVC (%)**		**6MWD (m)**	
	**P**	**r**	**P**	**r**	**P**	**r**	**P**	**r**	**P**	**r**	**P**	**r**
Age (Year)	-	-	0.755^†^	0.058	0.391^¶^	-0.160	0.441^¶^	-0.144	0.599^†^	-0.098	0.800^¶^	0.047
BMI (kg/m2)	0.755^†^	0.058	-	-	0.419^¶^	-0.151	0.820^¶^	0.043	0.338^†^	-0.178	0.101^¶^	-0.300
FEV1 (L)	0.391^¶^	-0.160	0.419^¶^	-0.151	-	-	-	-	-	-	0.298^¶^	0.193
FVC (L)	0.441^¶^	-0.144	0.820^¶^	0.043	-	-	-	-	-	-	0.544^¶^	0.113
FEV1/FVC (%)	0.599^†^	0.098	0.338^†^	-0.178	-	-	-	-	-	-	0.202^¶^	0.236
6MWD (m)	0.800^¶^	0.047	0.101^¶^	-0.300	0.298^¶^	0.193	0.544^¶^	0.113	0.202^¶^	0.236	-	-

6MWD: 6-minute walking distance; FEV1: forced expiratory volume in the first second; FVC: forced vital capacity; ¶: Spearman’s non-parametric test; †: Pearson’s test.

Table 3 examines the correlation of age, body mass index, FVC, FEV1, FEV1/FVC, and 6MWD by gender subgroups. Based on the results, there is no relationship between spirometric indices and 6MWD in gender subgroups.

**Table 3:** Correlation of age, body mass index, FEV1, FVC, FEV1/FVC, and 6MWD by gender subgroups

**Characteristic **	**Gender**											
	**Female**						**Male**					
	**6MWD (m)**		**FVC (L)**		**FEV1/FVC (%)**		**6MWD (m)**		**FVC (L)**		**FEV1/FVC (%)**	
	**P**	**r**	**P**	**r**	**P**	**r**	**P**	**r**	**P**	**r**	**P**	**r**
Age (Year)	0.859^¶^	-0.050	0.732^¶^	0.097	0.486^†^	-0.195	0.603^¶^	0.141	0.062^¶^	-0.477	0.781^†^	0.076
BMI (kg/m2)	0.420^¶^	-0.225	0.771^¶^	0.082	0.920^†^	0.029	0.052^¶^	-0.494	0.770^¶^	0.079	0.084^†^	-0.446
FEV1 (L)	0.820^¶^	0.064	-	-	-	-	0.307^¶^	0.273	-	-	-	-
FVC (L)	0.879^¶^	0.043	-	-	-	-	0.632^¶^	0.130	-	-	-	-
FEV1/FVC (%)	0.850^¶^	-0.054	-	-	-	-	0.059^¶^	0.482	-	-	-	-
6MWD(m)	-	-	0.879^¶^	0.043	0.850^¶^	-0.054	-	-	0.632^¶^	0.130	0.059^¶^	0.482

6MWD: 6-minute walking distance; FEV1: forced expiratory volume in the first second; FVC: forced vital capacity; ¶: Spearman’s non-parametric test; †: Pearson’s test.

## Discussion

 COPD patients are susceptible to different significant pulmonary and extrapulmonary complications, including airflow limitation, reduced physical activity, reduced weight, depression, and cardiovascular diseases.^12-14^ Therefore, close monitoring of these patients in terms of exercise tolerance is necessary for a better understanding of their prognosis. The aim of this study was to investigate the correlation between PFT and 6MWD tests in lung resection candidates with local lung diseases. The 6MWDtest is not widely used, and its predictive efficacy has not been established, hence the need for this study.Unlike previous studies that mainly focused on patients with systemic lung diseases, this study’s population had local lung diseases, and the most common indication for lung resection was hydatid cyst. However, no significant correlation was found between 6MWD and PFT parameters such as FEV1, FVC, and FEV1/FVC.

 PFT could be associated with complicaitons, including respiratory alkalosis brought on by excessive breathing, hypoxemia in a patient, fatigue, bronchospasm, paroxysmal coughing, chest discomfort, increased intracranial pressure, dizziness, and syncope.^15,16^ Therefore, using other techniques could pose patients with fewer compications.

 Previous studies have shown both supporting and conflicting results regarding the correlation between spirometry and the 6MWD test. Gontijo et al^17^ reported a weak positive correlation between FEV1/FVC and 6MWD in eutrophic and obese individuals. In contrast, our study found no significant correlation between these variables. In another cohort study by Rick et al^10^ on lung cancer patients with lung metastases who underwent lobectomy or pneumonectomy, PFT findings did not show a significant improvement, whereas 6MWD results showed a significant improvement after rehabilitation, suggesting a weak correlation between the two tests. The correlation between the 6MWD and spirometric parameters are mainly present in severe and very severe COPD patients.^14^ Furthermore, other reasons could be considered for the insignificant association between the two tests. Compared to the PFT, the 6MWD integrates different systems, including the respiratory, cardiovascular, circulation, and neuromuscular systems.^18,19^ Also, considering all the systems involved in exercise, once the pulmonary component is not severely distrupted, other systems could compensate. This explains the poor association between the 6MWD and the spirometric parameters in mild and moderate COPD.

 In contrast, Dinakar et al^20^ found a positive and statistically significant correlation between 6MWD outcomes and height, weight, FEV1 following bronchodilator treatment, FEV1, FVC, and FEV1/FVC in a study of 80 patients with COPD. Notably, there was no association between body mass index and 6MWD, while age was strongly negatively associated with 6MWD outcomes. Similarly, Agrawal et al^9^ examined the association between 6MWD test scores and spirometry in 130 individuals with COPD and found that FVC and FEV1 values were significantly correlated with 6MWD results. However, this study investigated patients with a generalized lung disease, which may explain the difference in results compared to the study by Dinakar et al on patients with COPD.

 Despite these variations, other studies have also shown that the 6MWD can be a valuable tool for preoperative assessment of surgery outcomes in patients with local pulmonary diseases. Rick et al^10^ assessed the predictive value of 6MWD in lung cancer patients who required lobectomy and reported that the 6MWD is a valuable tool for evaluating perioperative risk in lobectomy candidates. Similarly, Keeratichananont et al^21^ investigated the value of the preoperative 6MWD test in predicting the development of postoperative pulmonary complications in patients undergoing thoracic surgery and found that 6MWD was equivalent to FEV1 in predicting the likelihood of pulmonary complications after thoracic surgery.

## Conclusion

 Based on our research findings, we observed no association between the 6MWD and spirometry parameters. Despite its lower cost and simplicity compared to PFT, the 6MWD test is not a promising alternative. Therefore, we recommend that surgeons, especially cardiothoracic surgeons, refrain from relying on the 6MWD as a prognostic parameter for assessing patients’ respiratory function for lung resection, pending further robust evidence. We also suggest conducting additional research with larger sample sizes and diverse populations to strengthen the evidence base.

## Acknowledgements

 The authors hereby express their gratitude to the Medical Research Center in the Faculty of Medicine of Mashhad University of Medical Sciences.

## Competing Interests

 There is no conflict of interest.

## Ethical Approval

 All patients provided an informed consent to participate in the study. This research was authorized by the ethics committee of Mashhad University of Medical Sciences [IR.MUMS.MEDICAL.REC.1398.705].
